# Worsening of Oral Health Parameters After Head and Neck Radiotherapy—Findings from a Multidisciplinary Head and Neck Tumor Board: A Prospective Observational Study

**DOI:** 10.3390/cancers18193211

**Published:** 2026-10-05

**Authors:** Cosimo Rupe, Gioele Gioco, Benedetta Corraro, Anna Schiavelli, Silvia Longo, Francesco Pastore, Mariangela Massaccesi, Maria Antonietta Gambacorta, Francesco Miccichè, Raffaella Castagnola, Jacopo Galli, Stefano Settimi, Luca Raffaelli, Alessandra Cassano, Massimo Cordaro, Carlo Lajolo

**Affiliations:** 1 Head and Neck Department, Fondazione Policlinico Universitario Agostino Gemelli—IRCCS, School of Dentistry, Università Cattolica del Sacro Cuore, Largo A. Gemelli, 8, 00168 Rome, Italy; cosimorupe@gmail.com (C.R.); gioele.gioco@unicatt.it (G.G.); benedettacorraro@gmail.com (B.C.); anna.schiavelli@gmail.com (A.S.); luca.raffaelli@unicatt.it (L.R.); massimo.cordaro@unicatt.it (M.C.); carlo.lajolo@unicatt.it (C.L.); 2 Department of Radiation Oncology, Fondazione Policlinico Universitario Agostino Gemelli—IRCCS, Institute of Radiology, Università Cattolica del Sacro Cuore, Largo A. Gemelli, 8, 00168 Rome, Italy; silvia.longo@policlinicogemelli.it (S.L.); francesco.pastore@policlinicogemelli.it (F.P.); mariangela.massaccesi@policlinicogemelli.it (M.M.); mariaantonietta.gambacorta@policlinicogemelli.it (M.A.G.); 3 Oncologic Radiotherapy, Fatebenefratelli Isola Tiberina-Gemelli Isola, 00186 Rome, Italy; francesco.micciche@unicatt.it; 4 Head and Neck Department, Fondazione Policlinico Universitario Agostino Gemelli—IRCCS, Institute of Otolaryngology, Università Cattolica del Sacro Cuore, Largo A. Gemelli, 8, 00168 Rome, Italy; jacopo.galli@unicatt.it (J.G.); stefano.settimi@unicatt.it (S.S.); 5 Department of Medical Oncology, Fondazione Policlinico Universitario Agostino Gemelli—IRCCS, Institute of Radiology, Università Cattolica del Sacro Cuore, Largo A. Gemelli, 8, 00168 Rome, Italy; alessandra.cassano@unicatt.it

**Keywords:** radiotherapy, head and neck cancer, periodontitis, dental caries

## Abstract

**Simple Summary:**

This prospective cohort study aimed to evaluate changes in caries experience and periodontal status, as well as the need for dental extractions, in a cohort of patients treated with radiotherapy (RT) and chemoradiotherapy (RTCT) for head and neck cancer (HNC). In total, 113 patients were included (median follow-up: 44.77 months). Patients treated with RT experienced significant deterioration in dental and periodontal health.

**Abstract:**

Objectives: To evaluate (a) changes in caries experience, (b) changes in periodontal status, and (c) the need for dental extractions in a cohort of patients treated with radiotherapy (RT) and chemoradiotherapy (RTCT) for head and neck cancer (HNC). Methods: This prospective cohort study was approved by the local Ethics Committee (Ref. 22858/18) and registered at ClinicalTrials.gov (ID: NCT04009161). The study population consisted of patients with HNC who, prior to RT, underwent a dental examination followed by the elimination of oral foci of infection and were then enrolled in a follow-up program. The primary outcome was oral health worsening, defined as the onset of severe periodontitis and/or a DMFt score ≥ 13. Oral cavity RT dose was obtained contouring the ‘buccal mucosa’ organ at risk (OAR). Results: In total, 113 patients were included (median follow-up: 44.77 months). The sample mainly included oral, oropharyngeal, nasopharyngeal and laryngeal cancers (89/113, 78.8%). Salivary glands, hypopharynx and other sites accounted for a minority of the cases (24/113, 21.2%). Seventeen patients (34.7%) experienced worsening of oral health status over time, which was associated with smoking (OR = 4.7, 95% CI: 1.27–17.37; *p* = 0.02). The DMFt index increased (difference: 4.08; 95% CI: 1.60–6.56; *p* = 0.001), and this increase was associated with smoking (β = 2.05, 95% CI: 0.27–3.83; *p* = 0.02). Although worsening of periodontitis stage was not statistically significant (*p* = 0.35), localized deterioration was observed, with an increase in sites with PPD ≥ 5 mm (*p* = 0.02). Forty-nine patients (43.4%) experienced tooth loss. Mean tooth loss was 2.12 teeth per patient (*p* = 0.05), primarily due to caries (48.5%) and periodontitis (48.1%). Independent risk factors for tooth loss were age (*p* = 0.025) and an RT dose > 60 Gy to the ‘buccal mucosa’ OAR (*p* = 0.023). Conclusions: Patients treated with RT experienced significant deterioration in dental and periodontal health, and oral cavity dosimetric parameters might be associated with tooth loss, although this association requires confirmation in further studies.

## 1. Introduction

Head and neck cancers (HNCs) are a heterogeneous group of malignancies involving the oral cavity, pharynx, larynx, and salivary glands. They are among the most common malignancies worldwide, ranking seventh in global incidence [1].

Treatment is often multimodal and may involve surgery, radiotherapy (RT), and chemotherapy, frequently in combination as chemoradiotherapy [2]. Improved patient survival has led to growing interest in assessing and improving quality of life in HNC patients, with particular attention to oral and dental health [3,4], considering that HNC patients often show deterioration in their oral health status [5].

In particular, RT, administered in daily fractions at a total dose of 50–70 Gy, can cause several oral complications, such as oral mucositis [6], hyposalivation, xerostomia [7], dysgeusia, reduced mouth opening [8], and osteoradionecrosis (ORN) [9,10,11], but it may also impair dental health, since RT has been associated with caries and periodontitis, and subsequent tooth loss.

These associations may share common biological mechanisms. A major driver of worsening oral health in irradiated patients may be the loss of the protective effects of saliva. Furthermore, the use of RT in the head and neck region causes changes in the oral microbiome, with a shift to periodontal disease-associated flora.

Radiation-related caries (RC) are common adverse events after RT, as they are estimated to affect approximately 30% of patients and might cause serious damage to the oral health of HNC patients, especially when they lead to endodontic diseases, tooth fracture, tooth loss, and ORN [12].

Although the association between RT and dental caries is well known, a recent systematic review [13] revealed great heterogeneity among studies, an incomplete understanding of the pathogenesis of RC, and a relative paucity of data regarding modern RT techniques (i.e., intensity-modulated radiation therapy, IMRT), highlighting the need for well-designed prospective studies to allow a thorough analysis of the various factors related to dental caries in irradiated HNC patients.

On the other hand, RT is also thought to increase the incidence and severity of periodontitis via different pathways: hyposalivation might result in alterations in the oral microbiome [10,14], and radiation-induced fibrosis, reduced tissue perfusion and bone alterations might result in an impaired immune response to microbial challenges [15], thus resulting in clinical attachment loss.

Nevertheless, the association between RT and periodontitis has only recently been investigated, and the available evidence is scarce. Few studies have shown the worsening of periodontitis after older conventional modalities of RT [16], while only one prospective study investigated the impact of IMRT on the periodontal status of a large sample of patients, revealing modest and somewhat contradictory changes [17].

Although the detrimental effects of RT on oral health may significantly impair the quality of life of HNC patients, they have rarely been investigated, and the available evidence lacks prospective studies assessing the changes in the oral health of HNC patients who received a dental evaluation before RT and were enrolled in a follow-up program.

In this regard, prospective evidence may help to quantify the extent of dental and periodontal complications after RT, providing further insight into this poorly investigated topic.

In this context, this prospective study aimed to:

- Evaluate the worsening of oral health conditions after RT, with a minimum follow-up of 2 years (primary outcome).
- Assess risk factors for the deterioration of oral conditions in patients undergoing head and neck RT.
- Evaluate the incidence of tooth loss in patients enrolled in a follow-up program after RT.
- Evaluate the reasons for extractions after RT.

## 2. Materials and Methods

### 2.1. Study Design

This was a prospective observational cohort study reported according to the STROBE guidelines.

### 2.2. Setting

The study was conducted in accordance with the Declaration of Helsinki, and all patients provided written informed consent. The protocol was approved by the Ethics Committee of the Università Cattolica del Sacro Cuore (Ref. 22858/18) and registered on ClinicalTrials.gov (ID: NCT04009161, 5 July 2019). This study presents data derived from a cohort enrolled in a previously published cross-sectional study [18]. The included patients were diagnosed with HNC and had undergone an oral health assessment and clearance of oral foci prior to RT at the Oral Medicine outpatient clinic of Fondazione Policlinico Universitario A. Gemelli—IRCCS. Patients were visited between March 2017 and August 2021 and consecutively recruited for this study.

### 2.3. Inclusion Criteria

All patients who had received a diagnosis of HNC, had undergone a dental evaluation and elimination of oral foci prior to RT, and were subsequently enrolled in a follow-up protocol were included in the present study. All HNC cancers were included, including unknown primary, middle ear, paranasal sinuses.

### 2.4. Exclusion Criteria

At baseline, patients in whom an adequate clinical assessment of periodontal status could not be performed (individuals with sequelae of major oncologic surgery incompatible with the dental procedures necessary for diagnosis), edentulous patients, palliative RT, and patients who had already undergone RT in the head and neck region were excluded. Furthermore, patients who had not completed at least 2 years of follow-up after completion of RT were excluded from the present study.

### 2.5. Study Procedures

All patients were examined prior to RT with the support of an orthopantomogram (OPT). Demographic and medical history data were recorded, with particular attention to the patient’s oncologic history (tumor location, histological type, staging, previous oncological treatments) and exposure to risk factors for oncologic disease or for the dental diseases investigated (smoking, diabetes).

HNC were staged according to the eighth edition of the American Joint Committee on Cancer (AJCC) Staging Manual, Head and Neck Section [19].

A clinical evaluation of the following parameters was subsequently performed: presence of carious lesions and DMFt (Decayed, Missing, Filled teeth) score, periodontal status, and maximum mouth opening (MMO).

The DMFt index is the key measure of caries experience in dental epidemiology [20]. It sums the number of decayed teeth, missing teeth due to caries, and filled teeth in the permanent dentition.

Caries was diagnosed through clinical examination using a mouth mirror and a dental probe. In cases of uncertainty, the diagnosis was supported by intraoral radiography (periapical or bitewing with a Rinn holder), which was performed using film holders (Dentsply Sirona, Rome, Italy). When an endodontic or periodontal lesion needed to be diagnosed, a periapical radiograph was taken. Only caries involving dentin were included in the DMFt score. This diagnostic protocol was consistently maintained throughout the study period at every follow-up visit.

Periodontitis was assessed through a comprehensive periodontal examination by a single calibrated operator (C.L.) with more than ten years of experience in periodontology, using an NCP15 periodontal probe. The main periodontal parameters were recorded (i.e., probing pocket depth (PPD), gingival recession (REC), clinical attachment loss (CAL), furcation involvement (FI), tooth mobility, full-mouth plaque score (FMPS), and full-mouth bleeding score (FMBS)). Further details about the diagnostic procedures were reported in the baseline cross-sectional study [21].

Prior to data collection, intra-examiner calibration was performed by repeating periodontal measurements in a subset of patients after a 1-week interval. Reproducibility was assessed using ICC for probing depth measurements, showing excellent agreement (ICC: 0.90; 95% CI 0.85; 0.93), thereby supporting the reliability of the periodontal measurements.

Following the screening visit, patients underwent dental extractions that were judged to be clinically necessary, according to a previously published protocol [22]. Briefly, teeth were extracted in cases of untreatable periodontal, endodontic, and carious lesions. Similarly, third molars affected by pericoronitis were extracted.

After the removal of oral foci, periodontal conditions and DMFt were recording, representing the baseline assessment of the study. Then, patients underwent RT and were subsequently followed at regular intervals. Patients were evaluated 45, 90, and 180 days after RT. Patients were subsequently examined semiannually. During follow-up visits, oral conditions were re-evaluated.

RT was delivered using the volumetric-modulated arc radiotherapy (VMAT) technique with a linear accelerator, and treatment was administered in five daily fractions per week for 6–7 weeks.

The prescribed RT dose to the treatment volume of each patient was recorded. Post-RT, the treatment plans of the included patients were reviewed, and the RT dose to the oral cavity was calculated via the Eclipse Treatment Planning System program (Varian Medical System, Palo Alto, CA, USA).

In particular, the contouring of the ‘oral cavity’ refers to the prospectively contoured ‘buccal mucosa’ organ at risk (OAR), as defined by Brouwer et al. [23]. The buccal mucosa was contoured as a predefined OAR, and a retrospective ‘oral cavity’ contour was not performed to avoid heterogeneity in structure definition and reduce measurement variability. The buccal mucosa was contoured as the mucosal structure lateral to the mandible and maxilla. Anatomical boundaries were defined as follows: cranially, by the bottom of the maxillary sinus; caudally, by the upper edge of the teeth socket; anteriorly, by the lips/teeth; posteriorly, by the medial pterygoid muscle; laterally, by the buccal fat; and medially, by the outer surface of the mandible and maxilla and adjacent oral cavity/oropharyngeal structures. Dose–volume histogram (DVH) parameters for the buccal mucosa were extracted from the clinical treatment plans. The minimum dose (Dmin) was recorded as the lowest dose within the contoured buccal mucosa structure as derived from the DVH; the mean dose (Dmean) and maximum dose (Dmax) were also collected.

Minimum, mean, and maximum oral cavity dosimetric parameters were selected as simple descriptors of the dose distribution, with mean and maximum dose reflecting overall mucosal exposure and focal high-dose regions, respectively, in line with recent evidence supporting the association between oral cavity dose-volume parameters and radiation-induced oral mucositis [24].

Every six months, patients also underwent professional oral hygiene sessions, which involved the mechanical removal of plaque and calculus. They received instruction on home oral hygiene with personalized advice tailored to their individual needs. In cases of severe periodontitis, supportive periodontal care was provided every 3 months.

At each follow-up visit, the clinical evaluation was repeated, following the same procedures described above.

All dental conditions (i.e., caries, endodontic diseases, periodontitis, and tooth fractures) were diagnosed and treated according to the clinical needs of the included patients.

### 2.6. Outcomes

#### 2.6.1. Changes in Oral Health (OH) Status

The oral health (OH) parameter was defined as a dichotomous variable, and the DMFt index and periodontal staging were used to define the OH status. OH was defined as “poor” in the case of a DMFt score ≥ 13 and/or stage III or IV periodontitis and “good” only in the case of values below these thresholds for each of these variables. A DMFt score of 13 was chosen as the cutoff to define good oral health, as it has been reported as the average DMFt value in high-income countries [25]. Stages III and IV periodontitis were selected as cutoff values because they define “severe” periodontitis according to the 2017 classification of periodontal diseases [26].

#### 2.6.2. Hyposalivation and Mouth Opening

The stimulated salivary flow rate was assessed via the spitting method. Salivary secretion was stimulated by applying a solution of 2% citric acid to the sides of the tongue at intervals of 30 s. Patients were instructed to collect saliva for 5 min in a graded tube. Salivary flow < 0.4 mL/min was defined as hyposalivation [27]. Reduced maximum mouth opening (MMO) was reclassified to identify severe trismus according to the following cutoff: MMO ≤ 25 mm [28].

### 2.7. Statistical Analysis

The sample size was calculated using a z test for proportional power analysis. Assuming a worsening of oral health conditions in 20% of patients (with a precision of 5% and 90% power) and using 2% from a previous study [29] as the reference value, a sample size of 62 patients was obtained. This value was increased by 40% to account for potential drop-outs. Consequently, a final sample size of 103 was deemed necessary. This very high drop-out rate was estimated considering the poor prognosis of the HNC patients included in the study.

Qualitative variables were described using absolute frequencies and percentages. Quantitative variables were summarized as the means and standard deviations (SDs) for normally distributed data or as medians and percentiles for nonnormally distributed data.

The Shapiro–Wilk test was used to assess the normal distribution of quantitative variables. The Mann—Whitney U test and the Kruskal—Wallis test were used to compare continuous variables with nonparametric distributions, whereas parametric variables were analyzed via ANOVA. Correlation analyses were performed to evaluate the presence of any risk factors other than RT for deterioration in oral health. Pearson’s χ^2^ test and Fisher’s exact test were used to compare discontinuous variables between groups.

The difference between oral health conditions at baseline and those observed at the final follow-up was evaluated only for patients with baseline good OH. Univariate analysis was conducted to identify risk factors associated with changes in oral health. The identified risk factors were subsequently included in a stepwise logistic regression analysis to identify independent predictors of changes in oral health. The same analysis was performed to assess changes in the severity of periodontitis (i.e., changes in stage) over time.

The difference in DMFt values over time was assessed using Student’s *t* test. Similarly, the differences in the mean PPD and the number of pockets ≥ 5 mm over time were calculated.

Predictors for changes in the DMFt index were assessed via multiple linear regression, sequentially excluding nonsignificant risk factors (*p* > 0.05). The same analysis was performed to evaluate risk factors associated with an increase in PPD and in the number of pockets ≥ 5 mm.

Tooth survival was assessed at the tooth level using Kaplan–Meier analysis. Time zero was defined as the pre-radiotherapy dental assessment performed after removal of oral foci, and follow-up time was calculated from this assessment to tooth extraction or censoring at the last available follow-up visit. Because multiple teeth were nested within the same patient, within-patient clustering was accounted for using a cluster-robust variance estimator, with patient identification number specified as the clustering variable. Cluster-robust 95% confidence intervals were calculated for the survival estimates.

Logistic regression analysis was performed to identify any risk factors for tooth loss, as well as for hyposalivation and reduced MMO.

Adherence to the secondary-prevention protocol was used as a confounder in the regression models for OH worsening, DMFt changes, periodontal changes and tooth loss.

Furthermore, to evaluate the existence of any selection bias, the baseline data of the dropouts were compared to the data of the other patients with follow-up.

The statistical analysis was performed entirely via the Statistical Package for the Social Sciences software (IBM Corp. Released 2017. IBM SPSS Statistics for Apple, Version 25.0. Armonk, NY: IBM Corp).

## 3. Results

During the study period, 213 patients were evaluated and examined. At baseline, 18 patients were excluded because their oral conditions did not allow a proper diagnosis. After baseline examination, 8 patients were excluded due to edentulism. Therefore, the initial study sample consisted of 187 patients.

During the study, 74 patients (39.6% of the sample) dropped out: 56 patients died during the observation period (29.9%), 15 were unwilling to return to the clinic to complete the follow-up (residing outside the region or otherwise unavailable), and 3 patients did not respond to contact attempts for follow-up completion.

**Figure 1:**
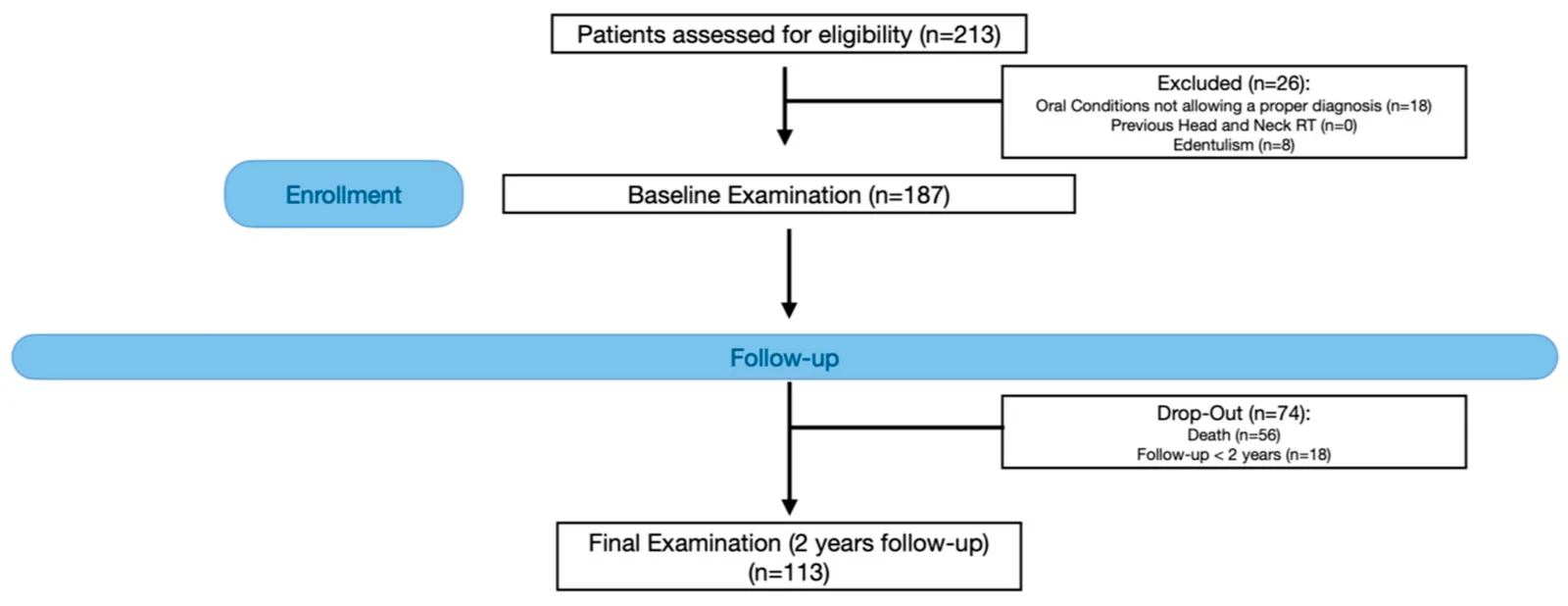
STROBE flowchart of the study.

The study flowchart is reported in Figure 1.

The final sample included 113 patients (35 women and 78 men, 31% and 69%, respectively, of the total sample) diagnosed with HNC, with a mean age of 57.2 years (SD: 11.43; range: 22–84), with a median follow-up of 44.77 months (range: 24.33–95.93). Eighty-three patients (73.45%) showed good adherence to the secondary-prevention protocol. Adherence to the secondary-prevention protocol did not have a statistically significant influence on the results of the multivariate analyses.

The mean RT dose delivered to the neoplastic site was 67.65 Gy (SD: 3.50; range: 50–72).

The most frequent tumor sites were oropharynx (35/113, 30.9%), oral cavity (19/113, 16.8%), nasopharynx (19/113, 16.8%) and laryngeal cancers (16/113, 14.2%). Salivary glands (11/113, 9.7%), hypopharynx (3/113, 2.7%) and other sites (10/113, 8.8%) accounted for a minority of the cases.

Most patients were affected by squamous cell carcinoma (SCC; 97/113, 85.8%). The most frequent tumor stages at diagnosis were stage 4 (48/113, 42.5%) and stage 3 (34/113, 30.1%). Chemotherapy was administered to 62.8% of the patients (71/113), while 37.2% underwent surgical intervention (42/113). A total of 64 patients (56.6%) were smokers, and 6.2% presented with diabetes (7/113).

The baseline characteristics of the patients who completed the follow-up were compared to the dropouts. The results of this analysis are available as Supplementary Materials. Tumor stage 4 and smoking were more frequent in dropouts, as well as severe periodontitis. Age and baseline DMFt were significantly higher.

### 3.1. Oral Health Deterioration

At baseline, 64 patients were classified as having poor OH, whereas 49 had good OH. Among the 49 patients with good OH at baseline, 17 (34.7%) experienced deterioration of their oral health status.

The results of the univariate analysis for OH worsening are presented in Table 1. Smoking and Dmin > 15 Gy were statistically significant.

The participant-level risk of OH worsening over the observation period was significantly influenced by smoking (OR = 4.7, 95% CI: 1.27–17.37; *p* = 0.02).

### 3.2. Analysis of Tooth Loss

The longitudinal analysis revealed a significant increase in the number of teeth lost between baseline and follow-up, with a mean tooth loss of 2.12 teeth per patient (*p* = 0.05). Overall, 239/2513 teeth were extracted, accounting for 9.51% of the total.

The distribution of causes for tooth loss was as follows: advanced caries (48.5%), periodontitis (48.1%), and fractures (3.4%). No endodontic failures were reported.

The results of the univariate analysis for risk factors for tooth loss are reported in Table 1.

Multivariate logistic regression analysis revealed that the following clinically relevant factors were independently associated with tooth loss during the follow-up period: age (OR: 1.042; 95% CI: 1.005–1.081; *p* = 0.025) and Dmax > 60 Gy to the oral cavity (buccal mucosa OAR, OR: 2.749; 95% CI: 1.148–6.585; *p* = 0.023).

**Figure 2:**
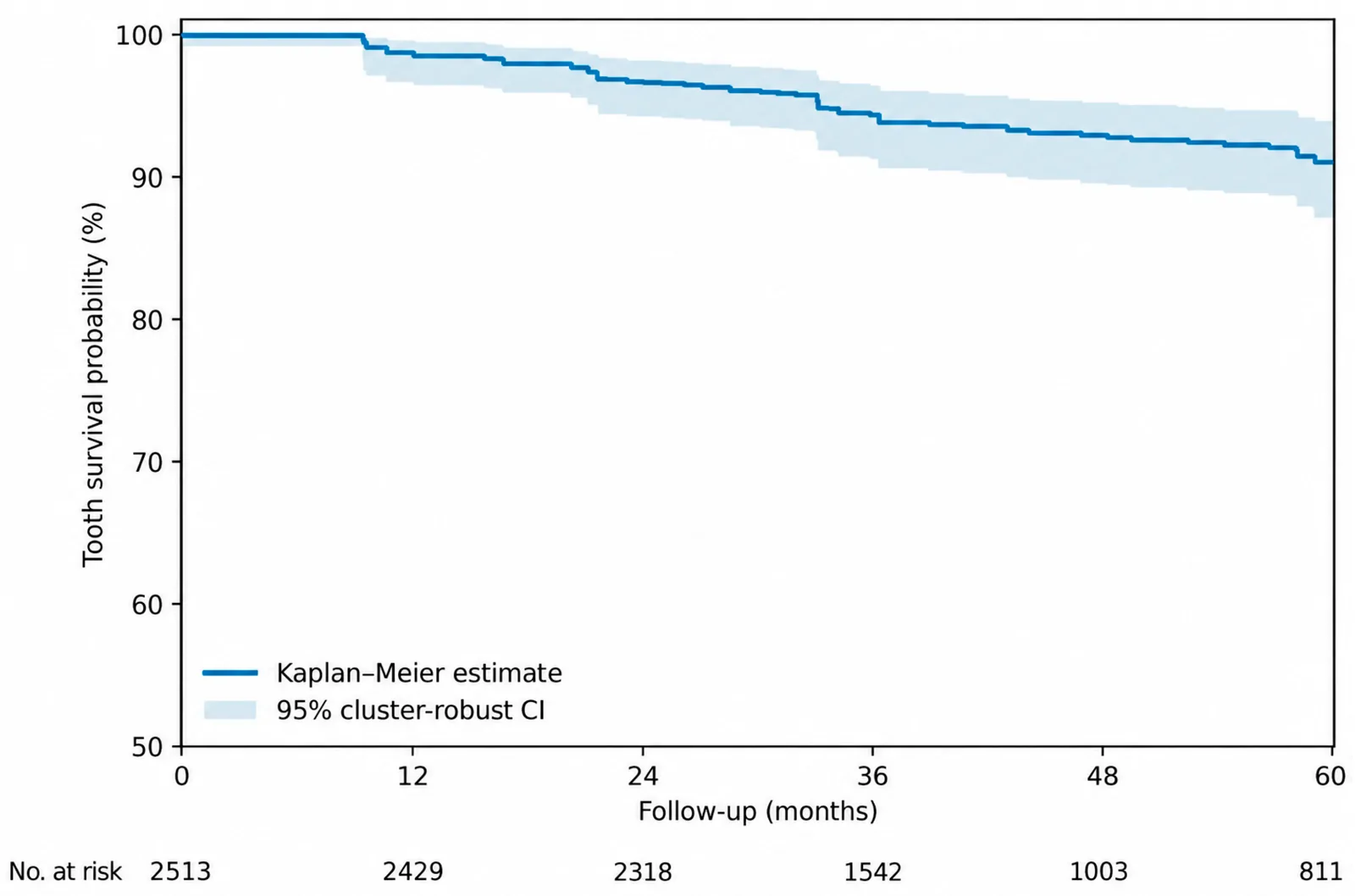
Cumulative survival rate (Kaplan–Meier curve for tooth survival). Time zero was defined as the pre-radiotherapy dental assessment performed after removal of oral foci. Shaded areas represent 95% confidence intervals calculated using a cluster-robust variance estimator to account for multiple teeth belonging to the same patient. The numbers below the x-axis indicate the number of teeth at risk at each time point.

The Kaplan–Meier curve for tooth survival is shown in Figure 2. A limited number of teeth were extracted during the first 24 months of follow-up. After 24 months, tooth survival began to decrease, reaching a cumulative survival rate below 80%, after approximately 72 months of follow-up.

### 3.3. Changes in DMFt and Periodontal Conditions

Table 2 shows the changes in the DMFt score and periodontal parameters.

The overall DMFt index increased significantly from baseline (15.5 ± 7.44) to follow-up (19. 61 ± 8.27). The statistical comparison between the two groups (baseline vs. follow-up) revealed a mean difference of 4.08 (95% CI: 1.60–6.56; *p* = 0.001).

Linear regression analysis showed that smoking habit (β = 2.05, 95% CI: 0.27–3.83, *p* = 0.02) was the only significant predictor of an increase in the DMFt index.

Compared with baseline, periodontal conditions significantly worsened, with an increase in the number of pockets ≥ 5 mm (3.28, 95% CI: 0.52–6.05, *p* = 0.02). Statistical analysis did not identify any specific risk factors for worsening of the mean PPD or for an increase in the number of pockets ≥ 5 mm. Furthermore, 8 patients progressed from nonsevere to severe periodontitis (*p* = 0.35).

### 3.4. Analysis of Reduced MMO and Hyposalivation

MMO decreased from 41.04 mm to 36.99 mm (difference: 4.05, 95% CI 2.86–5.24) and 16 patients developed trismus (MMO ≤ 25 mm) at the final follow-up. No correlation was found between oral health outcomes and a reduction in MMO.

Forty-three patients developed hyposalivation, but this finding was not significantly associated with worsening oral health conditions.

Table 3 shows the results of univariate analysis for hyposalivation and reduced MMO. No specific risk factors were identified for these outcomes.

## 4. Discussion

Although the effects of RT on oral health have been widely described, they have been less frequently investigated in prospective studies. The present study provides a long-term evaluation of oral health status in a cohort of irradiated HNC patients. The primary outcome data revealed a significant worsening of oral health: 17/49 initially healthy patients (34.7%) experienced deterioration of their condition compared with baseline.

Smoking emerged as the only significant risk factor for OH worsening (OR = 4.7, 95% CI: 1.27–17.37; *p* = 0.02). This result supports evidence of the negative role of tobacco use in the progression of dental diseases, likely through mechanisms altering the oral microenvironment and reducing local immune defenses [30,31,32].

The DMFt index and periodontitis were combined into a single endpoint to quantify the severity of both diseases and provide an integrated assessment, as previously proposed [5,21]. However, caries and periodontitis are multifactorial diseases with distinct biological behaviors, despite sharing a bacterial etiology. Therefore, the two diseases were also analyzed separately.

The deterioration in oral health status was quantitatively demonstrated by a significant increase in the DMFt index between baseline and follow-up (difference: 4.08; 95% CI: 1.60–6.56; *p* = 0.001). Radiation-related caries (RC) has a multifactorial etiology. Irradiation may cause both direct and indirect damage, with direct effects including reduced tooth microhardness and alterations at the enamel–dentin junction, leading to cracks and enamel delamination [33]. Indirect effects include hyposalivation, which impairs the self-cleansing function of saliva and may alter the oral microbiota [12]. These findings align with the literature showing that RT increases the risk of caries [13,34,35].

In particular, Moore et al. [36] reported a surprisingly similar finding, with 3.7 carious teeth per patients with caries in a 1-year prospective study conducted in the era of modern RT, while Brennan et al. [37] reported an increase of 4.29 carious teeth, slightly higher than the present cohort.

Smoking was confirmed to be a significant risk factor for an increase in RC (*p* = 0.02).

Furthermore, 8 patients progressed from nonsevere to severe periodontitis. This change was clinically relevant but not statistically significant (*p* = 0.35). The limited number of cases showing stage progression may be attributed to two main factors. First, most patients were already classified as stage IV at the initial assessment, which precluded further stage worsening. Second, staging is less sensitive to mild periodontal deterioration than other clinical parameters, such as probing pocket depth (PPD). PPD may capture smaller changes over time more effectively. Indeed, periodontal parameters showed a significant increase in sites with PPD ≥ 5 mm (difference: 3.28; 95% CI: 0.52–6.05; *p* = 0.02), suggesting post-RT deterioration in periodontal health. This deterioration may be mediated by reduced salivation and alterations in the local immune response [35]. The stability of mean PPD (*p* = 0.141) may reflect localized rather than generalized damage, indicating that most periodontal sites remained healthy.

In the present cohort, marked and accelerated worsening of periodontal status was observed after RT, independent of other periodontal risk factors (diabetes, smoking, and poor hygiene). Progression was faster than that reported by Becker [37]. Becker noted that patients treated for periodontitis without supportive periodontal therapy lost an average of 0.22 teeth/year, twice the rate observed in maintained patients (0.11 teeth/year) [38]. Despite the absence of maintenance, Becker reported no significant changes in mean PPD, which remained approximately 3.7–3.9 mm during follow-up. This finding indicates that mean values may mask clinically relevant deterioration at critical sites. In RT-treated patients, worsening appears more rapid and widespread, suggesting that RT may represent an important cofactor in periodontitis progression. No significant risk factor emerged as a major contributor to periodontal deterioration. Diabetes was substantially underpowered (n = 7; 0 in the worsening group); therefore, its effect cannot be evaluated in this cohort.

Beyond statistical significance, the observed oral health deterioration should be considered clinically relevant. Irradiated patients experienced substantial tooth loss, a severe caries experience, and worsening periodontal conditions, particularly when the oral cavity received higher radiation doses. These findings have direct implications for patient management and underscore the need to test more effective preventive strategies before, during, and after treatment.

From a clinical perspective, these findings support better integration of OH preservation into the multidisciplinary management of HNC patients. RT planning should include careful consideration of oral cavity and dental radiation exposure. Future work should also explore dose constraints for oral structures and structured long-term preventive dental care before, during, and after treatment.

This correlation might be explained as follows: preclinical studies have shown that RT may increase local levels of matrix metalloproteinase (MMP) and interleukin-6 (IL-6) [39]. Both of these mediators are upregulated in periodontal patients and may contribute to periodontal breakdown.

Our results add to the literature describing periodontal breakdown after RT. Ammajan et al. [15] reported increased clinical attachment loss and gingival recession 6 months after RT. Our findings differ slightly from those of the OraRad study [17], in which periodontal damage after RT was manifested mainly as gingival recession. Nevertheless, gingival recession and probing pocket depth represent different manifestations of the same disease and may be influenced by periodontal phenotype.

Caries and periodontitis contributed similarly to tooth extraction. In the present sample, 43.4% of patients lost at least one tooth. Tooth loss was significantly associated with advanced age (OR: 1.042; 95% CI: 1.005–1.081; *p* = 0.025) and exposure to high radiation doses in the oral region (OR: 2.749; 95% CI: 1.148–6.585; *p* = 0.023).

Interestingly, no cases of endodontic failure were reported. This finding is consistent with the high success rate of primary root canal treatment in postirradiated patients when strict asepsis and modern techniques are used [40]. Successful endodontic treatment may reduce the need for tooth extraction and subsequent ORN [41].

It should be noted that the changes in DMFt and tooth loss should not be interpreted as independent findings. Because teeth extracted during follow-up contribute both to the number of teeth lost and to the Missing component of the DMFt index, the two outcomes are intrinsically and partially overlapping. The high rate of tooth loss after the onset of RC might be explained by the atypical pattern of these lesions: RCs affect cervical areas and lingual surfaces, with rapid progression to crown fractures [12].

Advanced age emerged as a significant risk factor for tooth loss, with an OR of 1.042 (95% CI: 1.005–1.081; *p* = 0.025). A Dmax > 60 Gy was strongly associated with tooth loss (*p* = 0.023). This finding is consistent with the negative impact of RT on the vitality of dental and periodontal tissues, which is likely mediated by mechanisms of vascular damage and tissue hypoxia [42].

Unlike OH worsening, tooth loss was associated with Dmax > 60 Gy, suggesting that focal high-dose exposure may be relevant to tooth loss. However, this finding should be considered exploratory because of the inconsistency across dose metrics and the number of comparisons performed.

One novel observation is that the tooth loss experience was related to the radiation dose absorbed by the oral cavity. This finding may have important clinical implications. Even in patients treated for extraoral neoplasms, radiation oncologists might consider planning strategies that limit unnecessary oral cavity exposure to better preserve long-term periodontal health.

A recent 2-year follow-up study reported tooth failure in 17.8% of patients [43], a higher proportion than that observed in the present sample (9.51%). This difference may be explained by the strict pre-RT dental screening protocol, which included extraction of most teeth with a poor prognosis. The secondary prevention protocols applied in our cohort may also have contributed.

An unexpected finding was that hyposalivation was not significantly associated with worsening oral health in this sample (*p* = 0.324). This may be related to the use of VMAT, which reduces radiation exposure to the salivary glands compared with conventional techniques. Only 38.1% of patients developed hyposalivation. This limited number may have reduced our ability to detect the effect of salivary dysfunction on oral health.

This finding is supported by recent evidence showing that post-radiotherapy caries development may be more closely associated with direct radiation exposure to the dentition and oral cavity than with salivary parameters alone, further supporting the multifactorial nature of oral health deterioration after head and neck RT [36,37].

Similarly, only a minority of patients (14.2%) developed significant mouth opening limitations. The low prevalence of patients affected by trismus may have prevented the detection of a statistically significant association with worsening oral health.

Furthermore, a lower proportion of OH worsening was observed among patients who underwent oncological surgery. This counterintuitive association was not statistically significant. It may reflect pre-treatment dental extractions, differences in baseline dental status, or confounding related to tumor site and RT field.

Oral health deterioration in some patients may not depend only on RT. Oncological disease itself may be a predisposing factor, as numerous studies have linked neoplastic disease to compromised dental and periodontal health [10,13,41,43,44]. This association may reflect the effects of oncological therapies on the oral microbiota and the ability to maintain adequate hygiene. Chronic systemic inflammation associated with neoplasms may also promote periodontal disease progression [39,43]. Surgical interventions add further complexity. Clinical case series [44] indicate that teeth adjacent to mandibular resection or osteotomy sites have a greater risk of loss or of requiring endodontic treatment. This creates an additional challenge for long-term oral rehabilitation. Understanding these interactions is important for developing targeted preventive strategies for high-risk patients.

### Strengths and Limitations

The present study has several strengths. First, it enrolled a large sample with a relatively long follow-up. Patients were managed by a multidisciplinary tumor board at a national referral center. Furthermore, this study is the first to prospectively apply the 2017 classification of periodontal diseases in an HNC population. The lack of standardized diagnostic criteria for periodontitis in previous studies may limit comparisons across studies. The present study may therefore provide a useful benchmark for future research on the impact of RT on periodontal status.

However, the study has several limitations. Potential confounders include inconsistent adherence to the secondary prevention protocol and the lack of data on dietary habits, obesity, and metabolic syndrome. An additional limitation concerns the statistical power of the worsening analysis. Although the original sample size calculation targeted 103 participants, only 49 patients with good OH at baseline were at risk of worsening according to the predefined dichotomous outcome, and only 17 worsening events were observed. Therefore, the study should be considered underpowered for this outcome. This limits statistical power and the precision of effect estimates, particularly in multivariable analyses. Another limitation is the high number of dropouts during follow-up. This is partly understandable given the characteristics of the study population. Patients with cancer often undergo complex and demanding treatment pathways that can interfere with regular study visits, and mortality is high. In our sample, the mortality rate was consistent with the existing literature [45]. This possibility was anticipated during study planning, and the sample size calculation assumed a dropout rate of up to 40%. Nevertheless, the high dropout rate may have introduced selection bias because patients with worse general conditions and/or more aggressive disease could be underrepresented in the final analysis. Estimates of long-term oral outcomes should therefore be interpreted with caution. Restricting the analysis to 2-year survivors may also introduce survivor and selection bias and limits the generalizability of the results, especially with regard to the Kaplan–Meier analysis.

A further limitation is that multiple univariate tests were used to inform the subsequent stepwise regression analysis without adjustment for multiple comparisons. This may have increased the risk of type I error. The known instability of stepwise variable selection should also be considered when interpreting the final model. Because oral health worsening was defined as a participant-level binary outcome, logistic regression was appropriate. However, this approach does not model the full trajectory of repeated measurements collected during follow-up. Finally, the limited number of OH worsening events may make the multivariable model susceptible to overfitting. Its estimates should therefore be interpreted with caution.

Furthermore, the dose–response analysis relied on DVH metrics extracted from the prospectively contoured buccal mucosa OAR, used here as a surrogate of oral cavity mucosal exposure. However, the buccal mucosa does not fully correspond anatomically to the dental and periodontal structures evaluated in the clinical oral assessment. Therefore, the reported dose parameters should be interpreted as indicators of regional oral cavity exposure rather than as direct estimates of the dose received by teeth or periodontal tissues. This spatial mismatch may have introduced exposure misclassification and may have attenuated or obscured potential associations between radiation dose and specific dental or periodontal outcomes. Future studies including dedicated contouring of dental arches and periodontal structures may provide a more accurate assessment of dose–response relationships for oral health outcomes.

Furthermore, periodontal assessments were performed by a single experienced examiner, and baseline intra-examiner calibration was conducted. However, periodic recalibration was not performed during the extended follow-up period. This should be acknowledged as a potential source of measurement variability in periodontal outcomes over time. Another limitation is the absence of a non-irradiated comparator group, which limits our ability to distinguish changes specifically attributable to irradiation from those related to other factors.

Another limitation of the present study is that it included only routine prophylactic protocols, which were only partly effective in counteracting oral health deterioration.

Future studies testing personalized protocols (i.e., bimonthly supportive periodontal care, fluoride treatments, and dietary protocols), with particular attention to elderly patients and those receiving high-dose RT, should be designed to reduce oral health worsening after RT.

## 5. Conclusions

The results of this study indicate a worsening of oral health in patients undergoing RT, particularly in terms of tooth loss, driven by both caries and periodontitis.

RT may represent a significant risk factor for the progression of periodontitis, although no dose–response relationship was found. Further studies are needed to better investigate the mechanisms supporting this finding.

Oral cavity dosimetric parameters appeared to be among the most clinically relevant predictors of tooth loss, suggesting that radiation exposure to this region may contribute to tooth loss, caries development, and periodontal worsening. These findings should be interpreted cautiously but may support future efforts to define dose constraints for oral structures and to strengthen preventive dental care protocols in HNC patients.

These results require confirmation in future studies conducted in different cohorts with longer follow-up. The complex interactions between RT, oral health, and individual variables (such as hygiene, diet, and comorbidities) require further investigation to delineate the pathogenic mechanisms more precisely and define effective prevention and management strategies.

## Figures and Tables

**Table 1:** Results of the univariate analysis for deterioration in OH. SD: standard deviation. Bold values highlight statistical significance. ^a^ Both surgery of the primary tumor and neck dissection.

Outcome		*Worsening of OH*	*No Worsening of OH*	Statistical Significance	*Tooth Loss*	No Tooth Loss	Statistical Significance
*Sex*	Man	11 (64.7%)	20 (62.5%)	χ^2^ Test—*p* = 0.566	36 (73.5%)	42 (65.6%)	χ^2^ Test—*p* = 0.416
	Woman	6 (35.3%)	12 (37.5%)		13 (26.5%)	22 (34.4%)	
	Total	17 (100%)	32 (100%)		49 (100%)	64 (100%)	
*Age (Years)*	Mean (Range, SD)	51.0 (25–67; SD: 11.7)	52.6 (22–71; SD: 13.03)	ANOVA—*p* = 0.663	59.9 (25–84; SD: 10.7)	55.0 (22–72; SD: 11.6)	**ANOVA—*p* = 0.023**
*Tumor Pathology*	SCC	14 (82.4%)	26 (81.3%)	χ^2^ Test—*p* = 0.364	43 (87.8%)	54 (84.4%)	χ^2^ Test—*p* = 0.701
	Other types	3 (17.6%)	6 (16.7%)		6 (12.2%)	10 (15.6%)	
	Total	17 (100%)	32 (100%)		49 (100%)	64 (100%)	
*Tumor Stage*	Stage 1	1 (6%)	2 (6.2%)	χ^2^ Test—*p* = 0.809	6 (12.2%)	5 (7.8%)	χ^2^ Test—*p* = 0.290
	Stage 2	4 (23.5%)	9 (28.1%)		5 (10.2%)	15 (23.4%)	
	Stage 3	4 (23.5%)	11 (34.4%)		15 (30.6%)	19 (29.7%)	
	Stage 4	8 (47%)	10 (31.3%)		23 (46.9%)	25 (39.1%)	
	Total	17 (100%)	32 (100%)		49 (100%)	64 (100%)	
*Tumor Site*	Hypopharynx	0 (-)	0 (-)	χ^2^ Test—*p* = 0.690	2 (4.1%)	1 (1.6%)	χ^2^ Test—*p* = 0.686
	Larynx	2 (11.8%)	4 (12.5%)		7 (14.3%)	9 (14.1%)	
	Oral cavity	2 (11.8%)	7 (21.8%)		8 (16.4%)	11 (17.2%)	
	Oropharynx	7 (41.2%)	9 (28.1%)		18 (36.7%)	17 (26.6%)	
	Nasopharynx	4 (23.5%)	6 (18.8%)		5 (10.2%)	14 (21.9%)	
	Salivary Glands	2 (11.8%)	3 (9.4%)		5 (10.2%)	6 (9.4%)	
	Other sites	0 (-)	3 (9.4%)		4 (8.2%)	6 (9.4%)	
	Total	17 (100%)	32 (100%)		49 (100%)	64 (100%)	
*Smoking*	Smokers	12 (70.6%)	10 (31.3%)	**χ^2^ Test—*p* = 0.010**	31 (63.3%)	33 (51.6%)	χ^2^ Test—*p* = 0.253
	Non-smokers	5 (29.4%)	22 (68.7%)		18 (36.7%)	31 (48.4%)	
	Total	17 (100%)	32 (100%)		49 (100%)	64 (100%)	
*Oncological surgery ^a^*	Performed	3 (17.7%)	15 (46.9%)	χ^2^ Test—*p* = 0.06	21 (42.9%)	21 (32.8%)	χ^2^ Test—*p* = 0.328
	Not Performed	14 (82.3%)	17 (53.1%)		28 (57.1%)	43 (67.2%)	
	Total	17 (100%)	32 (100%)		49 (100%)	64 (100%)	
*Chemotherapy*	Performed	13 (76.5%)	18 (56.3%)	χ^2^ Test—*p* = 0.219	29 (59.2%)	42 (65.6%)	χ^2^ Test—*p* = 0.557
	Not Performed	4 (23.5%)	14 (43.7%)		20 (40.8%)	22 (34.4%)	
	Total	17 (100%)	32 (100%)		49 (100%)	64 (100%)	
*Diabetes*	Yes	0 (-)	1 (3.1%)	χ^2^ Test—*p* = 0.653	3 (6.1%)	4 (6.3%)	χ^2^ Test—*p* = 1.000
	No	17 (100%)	31 (96.9%)		46 (93.9%)	60 (93.7%)	
	Total	17 (100%)	32 (100%)		49 (100%)	64 (100%)	
*Hyposalivation post-RT*	Yes	5 (29.4%)	13 (40.6%)	χ^2^ Test—*p* = 0.324	14 (28.6%)	29 (45.3%)	χ^2^ Test—*p* = 0.081
	No	12 (70.6%)	19 (59.4%)		35 (71.4%)	35 (54.7%)	
	Total	17 (100%)	32 (100%)		49 (100%)	64 (100%)	
*Decreased MMO*	Yes	2 (11.7%)	5 (15.6%)	χ^2^ Test—*p* = 1.00	7 (14.3%)	9 (14.1%)	χ^2^ Test—*p* = 1.000
	No	15 (88.3%)	27 (84.4%)		42 (85.7%)	55 (85.9%)	
	Total	17 (100%)	32 (100%)		49 (100%)	64 (100%)	
*Maximum oral dose (DMax)*	>60	14 (82.4%)	22 (68.7%)	χ^2^ Test—*p* = 0.250	39 (79.6%)	38 (59.4%)	**χ^2^ Test—*p* = 0.026**
	≤60	3 (17.6%)	10 (31.3%)		10 (20.4%)	26 (40.6%)	
	Total	17 (100%)	32 (100%)		49 (100%)	64 (100%)	
*Mean oral dose (DMean)*	>40	9 (52.9%)	15 (46.9%)	χ^2^ Test—*p* = 0.458	24 (49%)	26 (40.6%)	χ^2^ Test—*p* = 0.446
	≤40	8 (47.1%)	17 (53.1%)		25 (51%)	38 (59.4%)	
	Total	17 (100%)	32 (100%)		49 (100%)	64 (100%)	
*Minimum oral dose (DMin)*	>15	10 (58.8%)	9 (28.1%)	**χ^2^ Test—*p* = 0.009**	13 (26.5%)	22 (34.4%)	χ^2^ Test—*p* = 0.416
	≤15	7 (41.2%)	23 (71.9%)		36 (73.5%)	42 (65.6%)	
	Total	17 (100%)	32 (100%)		49 (100%)	64 (100%)	
*RT total dose*	Mean (Range, SD)	68.2 (60–70; SD: 2.8)	67.3 (60–72; SD:3.6)	ANOVA—*p* = 0.369	67.3 (60–70; SD: 3.4)	67.9 (50–72; SD: 3.6)	ANOVA—*p* = 0.313

**Table 2:** Changes in DMFt and periodontal parameters.

Outcome	Baseline Mean (±SD)	Follow-Up Mean (±SD)	Difference	95% CI	*p* Value
*Decayed teeth*	1.17 (±1.5)	1.61 (±3.13)	0.44	−0.2–1.08	0.176
*Missing teeth*	9.71 (±9.71)	11.83 (±9.06)	2.12	0.04–4.20	**0.05**
*Filled teeth*	4.65 (±4.11)	6.17 (±4.89)	1.52	0.35–2.71	**0.01**
*Overall DMFt*	15.53 (± 7.44)	19.61 (± 8.27)	4.08	1.60–6.56	**0.001**
*Mean PPD (mm)*	2.40	2.62	0.22	−0.07–0.52	0.141
*Sites with PPD ≥ 5 mm (n)*	4.20	7.48	3.28	0.52–6.05	**0.02**

Bold values highlight statistical significance.

**Table 3:** Results of the univariate analysis for hyposalivation and reduced maximum mouth opening. SD: standard deviation. ^a^ Both surgery of the primary tumor and neck dissection.

Outcome		*Hyposalivation*	No *Hyposalivation*	Statistical Significance	*Reduced MMO*	No *Reduced MMO*	Statistical Significance	Total
*Sex*	Man	28 (65.1%)	50 (71.4%)	χ^2^ Test—*p* = 0.533	12 (75.0%)	66 (68.0%)	χ^2^ Test—*p* = 0.571	78 (69.0%)
	Woman	15 (34.9%)	20 (28.6%)		4 (25.0%)	31 (32.0%)		35 (31.0%)
	Total	43 (100%)	70 (100%)		16 (100%)	97 (100%)		113 (100%)
*Age (Years)*	Mean (Range, SD)	56.56 (22–81, SD: 12.6)	57.46 (25–84, SD:10.7)	ANOVA—*p* = 0.687	58.6 (37–72, SD: 10.2)	56.9 (22–84, 11.6)	ANOVA—*p* = 0.409	57.2 (22–84; SD: 11.43)
*Tumor Pathology*	SCC	36 (51.4%)	61 (87.1%)	χ^2^ Test—*p* = 0.113	10 (62.5%)	87 (89.7%)	χ^2^ Test—*p* = 0.173	97 (85.8%)
	Other types	7 (48.6%)	9 (12.9%)		6 (37.5%)	10 (10.3%)		16 (14.2%)
	Total	43 (100%)	70 (100%)		16 (100%)	97 (100%)		113 (100%)
*Tumor stage*	Stage 1	2 (4.6%)	9 (12.9%)	χ^2^ Test—*p* = 0.567	2 (12.5%)	9 (9.3%)	χ^2^ Test—*p* = 0.594	11 (9.7%)
	Stage 2	8 (18.6%)	12 (17.1%)		2 (12.5%)	18 (18.6%)		20 (17.7%)
	Stage 3	13 (30.2%)	21 (30.0%)		6 (37.5%)	28 (28.9%)		34 (30.1%)
	Stage 4	20 (46.5%)	28 (40.0%)		6 (37.5%)	42 (43.3%)		48 (42.5%)
	Total	43 (100%)	70 (100%)		16 (100%)	97 (100%)		113 (100%)
*Tumor Site*	Hypopharynx	0 (0%)	3 (4.3%)	χ^2^ Test—*p* = 0.842	0 (0%)	3 (3.1%)	χ^2^ Test—*p* = 0.686	3 (2.7%)
	Larynx	7 (16.3%)	9 (12.9%)		1 (6.3%)	15 (15.5%)		16 (14.2%)
	Oral cavity	7 (16.3%)	12 (17.1%)		1 (6.3%)	18 (18.6%)		19 (16.8%)
	Oropharynx	14 (32.6%)	21 (30.0%)		6 (37.5%)	29 (29.9%)		35 (31.0%)
	Nasopharynx	7 (16.3%)	12 (17.1%)		3 (18.8%)	16 (16.5%)		19 (16.8%)
	Salivary Glands	3 (7.0%)	8 (11.4%)		3 (18.8%)	8 (8.2%)		11 (9.7%)
	Other sites	5 (11.6%)	5 (7.1%)		2 (12.6%)	8 (8.2%)		10 (8.9%)
	Total	43 (100%)	70 (100%)		16 (100%)	97 (100%)		113 (100%)
*Smoking*	Smokers	22 (51.2%)	42 (60.0%)	χ^2^ Test—*p* = 0.435	9 (56.2%)	55 (56.7%)	χ^2^ Test—*p* = 1.000	64 (56.6%)
	Non-smokers	21 (48.8%)	28 (40.0%)		7 (43.8%)	42 (43.3%)		49 (43.4%)
	Total	43 (100%)	70 (100%)		16 (100%)	97 (100%)		113 (100%)
*Oncological surgery ^a^*	Performed	12 (27.9%)	30 (42.9%)	χ^2^ Test—*p* = 0.160	6 (37.5%)	36 (37.1%)	χ^2^ Test—*p* = 1.000	42 (37.2%)
	Not Performed	31 (72.1%)	40 (57.1%)		10 (62.5%)	61 (62.9%)		71 (62.8%)
	Total	43 (100%)	70 (100%)		16 (100%)	97 (100%)		113 (100%)
*Chemotherapy*	Performed	31 (72.1%)	40 (57.1%)	χ^2^ Test—*p* = 0.160	13 (81.3%)	58 (59.8%)	χ^2^ Test—*p* = 0.161	71 (62.8%)
	Not Performed	12 (27.9%)	30 (42.9%)		3 (18.7%)	39 (40.2%)		42 (37.2%)
	Total	43 (100%)	70 (100%)		16 (100%)	97 (100%)		113 (100%)
*Diabetes*	Yes	2 (4.7%)	5 (7.1%)	χ^2^ Test—*p* = 0.707	1 (6.3%)	6 (6.2%)	χ^2^ Test—*p* = 1.000	7 (6.2%)
	No	41 (95.2%)	65 (92.9%)		15 (93.7%)	91 (93.8%)		106 (93.8%)
	Total	43 (100%)	70 (100%)		16 (100%)	97 (100%)		113 (100%)
*Maximum oral dose (Dmax)*	>60	28 (65.1%)	49 (70.0%)	χ^2^ Test—*p* = 0.679	13 (81.3%)	64 (66.0%)	χ^2^ Test—*p* = 0.263	77 (68.1%)
	≤60	15 (34.9%)	21 (30.0%)		3 (18.7%)	33 (34.0%)		36 (31.9%)
	Total	43 (100%)	70 (100%)		16 (100%)	97 (100%)		113 (100%)
*Mean oral dose (Dmean)*	>40	20 (46.5%)	30 (42.9%)	χ^2^ Test *p* = 0.846	7 (43.8%)	43 (44.3%)	χ^2^ Test—*p* = 1.000	50 (44.2%)
	≤40	23 (53.5%)	40 (57.1%)		9 (56.2%)	54 (55.6%)		63 (55.8%)
	Total	43 (100%)	70 (100%)		16 (100%)	97 (100%)		113 (100%)
*Minimum oral dose (Dmin)*	>15	15 (34.9%)	20 (28.6%)	χ^2^ Test *p* = 0.533	6 (37.5%)	29 (29.9%)	χ^2^ Test—*p* = 0.567	35 (31.0%)
	≤15	28 (65.1%)	50 (71.4%)		10 (62.5%)	68 (70.1%)		78 (69.0%)
	Total	43 (100%)	70 (100%)		16 (100%)	97 (100%)		113 (100%)
*Tooth loss*	Yes	14 (32.6%)	35 (50.0%)	χ^2^ Test *p* = 0.081	7 (43.8%)	42 (43.3%)	χ^2^ Test—*p* = 1.000	49 (43.3%)
	No	29 (67.4%)	35 (50.0%)		9 (56.2%)	55 (56.7%)		64 (56.6%)
	Total	43 (100%)	70 (100%)		16 (100%)	97 (100%)		113 (100%)
*OH change*	Yes	5 (11.6%)	12 (17.1%)	χ^2^ Test *p* = 0.589	2 (12.5%)	15 (15.5%)	χ^2^ Test—*p* = 1.000	17 (15.0%)
	No	38 (88.4%)	58 (82.9%)		14 (87.5%)	82 (84.5%)		96 (85.0%)
	Total	43 (100%)	70 (100%)		16 (100%)	97 (100%)		113 (100%)

## Data Availability

The data that support the findings of this study are available from the corresponding author, Raffaella Castagnola, upon reasonable request.

## Supplementary Materials

The following supporting information can be downloaded at: https://www.mdpi.com/article/10.3390/cancers18193211/s1, Table S1: Differences in baseline characteristics between completers and drop-outs. File S1: STROBE Statement—Checklist of items that should be included in reports of cohort studies.
