# Supplementary material for: Worsening of Oral Health Parameters After Head and Neck Radiotherapy—Findings from a Multidisciplinary Head and Neck Tumor Board: A Prospective Observational Study
Source: Cancers (Basel). 2026 Oct 5;18(19):3211. doi: 10.3390/cancers18193211 (PMC13664503; doi:10.3390/cancers18193211)
Supplement: Supplementary file 1 [file cancers-18-03211-s001.zip › Supplementary Table S1.pdf]

Table S1. *Results of the univariate analysis for deterioration in OH. SD: standard deviation.*

Outcome		Completers	Dropouts	Statistical significance
Sex	Man	78 (69%)	47 (63.5%)	χ2 Test —p=0.525
	Woman	35 (31%)	27 (36.6%)	
	Total	113 (100%)	74 (100%)	
Age (Years)	Mean (Range, SD)	57.1 (22-84; SD: 11.4)	63.1 (33-86; SD: 10.2)	**ANOVA – p= 0.0001**
Tumor Pathology	SCC	97 (85.8%)	68 (91.9%)	χ2 Test —p=0.701
	Other types	16 (14.2%)	6 (8.1%)	
	Total	113 (100%)	74 (100%)	
Tumor Stage	Stage 1	11 (9.7%)	1 (1.3%)	**χ2 Test —p=0.04**
	Stage 2	20 (17.7%)	9 (12.2%)	
	Stage 3	34 (30.1%)	25 (33.8%)	
	Stage 4	48 (42.5%)	39 (52.7%)	
	Total	113 (100%)	74 (100%)	
Tumor Site	Hypopharynx	3 (2.7%)	3 (4.1%)	χ2 Test —p=0.101
	Larynx	16 (14.6%)	23 (31.1%)	
	Oral cavity	19 (16.8%)	13 (17.6%)	
	Oropharynx	35 (30.9%)	15 (20.3%)	
	Nasopharynx	19 (16.8%)	10 (13.5%)	
	Salivary Glands	11 (9.7%)	3 (4.1%)	
	Other sites	10 (8.8%)	7 (9.5%)	
	Total	113 (100%)	74 (100%)	
Smoking	Smokers	64 (56.6%)	55 (74.3%)	**χ2 Test —p=0.019**
	Non-smokers	49 (43.4%)	19 (25.7%)	
	Total	113 (100%)	74 (100%)	
Oncological surgery	Performed	42 (37.2%)	31 (41.9%)	χ2 Test —p=0.543
	Not Performed	71 (62.8%)	43 (58.1%)	
	Total	113 (100%)	74 (100%)	
Chemotherapy	Performed	71 (62.8%)	41 (55.4%)	χ2 Test —p=0.361
	Not Performed	42 (37.2%)	33 (44.6%)	
	Total	113 (100%)	74 (100%)	
Diabetes	Yes	7 (6.2%)	5 (6.8%)	χ2 Test —p=1.000
	No	106 (93.8%)	69 (93.2%)	
	Total	113 (100%)	74 (100%)	
Stage of Periodontitis	No	21 (18.6%)	12 (16.2%)	**χ2 Test – p=0.001**
	Stage 1	17 (15%)	5 (6.8%)	
	Stage 2	26 (23.1%)	8 (10.8%)	

	Stage 3	20 (17.7%)	5 (6.8%)	
	Stage 4	29 (25.7%)	39 (52.7%)	
	Total	113 (100%)	74 (100%)	
Total DMFt	Mean (Range, SD)	15.53 (0-32; SD: 7.44)	19.46 (0-32; SD: 8.8)	**ANOVA – p= 0.013**
